# High preoperative serum sVCAM-1 concentration as a predictor of early ovarian cancer recurrence

**DOI:** 10.1186/s13048-020-00705-9

**Published:** 2020-09-15

**Authors:** Marina Jakimovska, Katarina Černe, Ivan Verdenik, Borut Kobal

**Affiliations:** 1 Department of Obstetrics and Gyaecology, University Medical Centre, Ljubljana, Slovenia; 2 Institute of Pharmacology and Experimental Toxicology, Faculty of medicine, University of Ljubljana, Ljubljana, Slovenia; 3 Faculty of medicine, University of Ljubljana, Ljubljana, Slovenia; 4 Department of Gynaecology and Obstetrics, Faculty of medicine, University of Ljubljana, Šlajmarjeva 3, SI-1000 Ljubljana, Slovenia

**Keywords:** sVCAM-, Ovarian cancer, Early recurrence

## Introduction

Epithelial Ovarian cancer (EOC) is the most lethal gynecological cancer and more than 70% of all cases are diagnosed at an advanced stage. It has been shown that not only complete cytoreductive surgery, but also tumor chemotherapy sensitivity is crucial in terms of relapse time. Even if approximately 80% of the women respond to treatment, the relapse is common although a complete clinical remission is obtained [1]. Most of the patient will relapse within 24 months of the treatment, which has unfortunately been unchanged in recent decades despite developments in chemo pharmacy [2]. Predicting risk of recurrence will allow patients to have a better quality of life and clinicians to better approach in ovarian cancer treatment.

Several tumour markers have been studied in the serum of ovarian cancer patients to provide better disease monitoring. One such protein has been Soluble Vascular Cell Adhesion Molecule-1 (sVCAM-1) [3]. Its elevated concentration is shown to be associated with tumour presence [4]. Expressed on activated endothelial and mesothelial cells VCAM-1 has been identified as an important mediator for adhesion of ovarian cancer cells to and invasion through the mesothelium [5].

In our previous study we evidenced the positive correlation of the sVCAM-1 concentration in ovarian cancer patient’s serum and ascites, which represents tumour microenvironment [3]. The aim of the current study was to analyse preoperative serum and ascites sVCAM- 1 concentration and to correlate them to ovarian cancer recurrence and survival.

## Materials and methods

### Study design

We conducted an observational cohort prospective study on patients with diagnosis of advanced stage (FIGO stage III or IV) primary EOC operated at Division of Gynecology and Obstetrics, University Medical Centre Ljubljana, Slovenia, between 2011 to 2013. The trial was approved by the National Medical Ethics Committee of Republic of Slovenia (Approval number 82/01/11).

### Inclusion and exclusion criteria

We considered as inclusion criteria: diagnosis of primary EOC with FIGO stage III or IV independently from tumor grading, absence of concomitant malignant neoplasms, patients that underwent primary-debulking surgery (PDS) or neoadjuvant chemotherapy (NACT) plus interval debulking surgery (IDS). We excluded all patients with diseases demonstrated to influence sVCAM-1 concentration (active inflammation, complicated diabetes, various autoimmune diseases), nonepithelial histologic type. All patients received and signed consent documentation about the research and analysis of their blood and ascites for the purposes of the research.

### Data collection and definition of groups

Clinical data was collected in a specific computer database during the various step of surgical/oncological treatment and follow-up. We collected data about patients’ age, FIGO stage, tumor grading, histologic type, and type of treatment (PDS or NACT+IDS).

For data analysis patients were split according to the disease progression in Group A (patients with disease progress or tumor relapse within 12 months of treatment completion) and Group B (patients with tumor relapse occuring more than 12 months after treatment completion). By completion of the treatment is meant the end of the last cycle of chemotherapy.

Follow up took place until 24 March 2016 or until patient death. The final patient in the study had surgery 19 December 2013. The minimum follow up period was 27 months, the maximum 58 months.

### Samples collection and analysis

Before operation four ml of peripheral blood were collected in a vacutianer, without anticoagulant or other additives and used for sVCAM-1 analysis. Twenty ml of ascites were aspirated into a sterile syringe at the beginning of each operation and immediately transferred into a conical tube and kept on ice until centrifugation at 1000 x *g* for 10 min at 4 °C. Serum was separated by centrifugation at 2000 x *g* for 15 min at 4 °C. Ascites supernatants and serum were stored in aliquots at − 80 °C. sVCAM-1 sample concentration was analyzed by means of flow cytometric bead-based assay and measured using a FlowCytomix Simplex Kit (eBio-science, Vienna). The kit consists of fluorescent microspheres (diameter: 4 μm, emission wavelength at 700 nm) coated with specific sVCAM-1antibodies raised.

### Statistical analysis

Data are presented as mean ± SD. The normality of distribution was tested with the Kolmogorov-Smirnov test. The Mann-Whitney test was used for non-normally distributed variables. We used a chi squared test for nominal variables and ANOVA for continuous variable such as age, which are normally distributed. ROC analysis was used to identify a cut-off value for sVCAM-1. Survival curves were calculated using Kaplan-Meier method. The association between sVCAM-1 and survival was investigated with Cox regression models, adjusting for age at surgery. A *p* of < 0.05 was considered significant. Statistical analysis was performed using software statistical package SPSS, version 21 (IBM Corp, Armonk, NY).

## Results

### Patients’ characteristics

During the study period a total of 123 patients with FIGO stage III and IV were reffered to the Division of Gynecology and Obstetrics, University Medical Centre Ljubljana. A total of 37 patients were included in the study based on inclusion and exclusion criteria and the consent to study participation. According to tumor relapse or disease progress within 12 months from treatment completion, a total of 20 patients were included in Group A (disease progress or tumor relapse within 12 months) and 17 patients in Group B (disease progress or tumor relapse more than 12 months). There was no statistically significant difference between the groups in disease histology, grade and stage. Group B were statistically significantly younger (*p* = 0.01), with a higher percentage of them treated with primary cytoreductive surgery (*p* = 0.01) compared with Group A. General features of included patients according to groups are reported in Table 1.

**Table 1:** Patient demographic and treatment characteristics

	Group A n	Group B n	All patients n
Number of patients	20	17	37
Age	63.3±10.8	53.9±10.6	59 ± 11,6
FIGO stage III	13	15	28
FIGO stage IV	7	2	9
Serouse tip	19	13	32
Endometrioid type	1	4	5
G1	2	3	5
G2	6	5	11
G3	12	9	21
Primary citoreductive operation	6	12	18
Neoadjuvant chemotherapy	14	5	19

### sVCAM-1 concentrations among groups

**Fig. 1:**
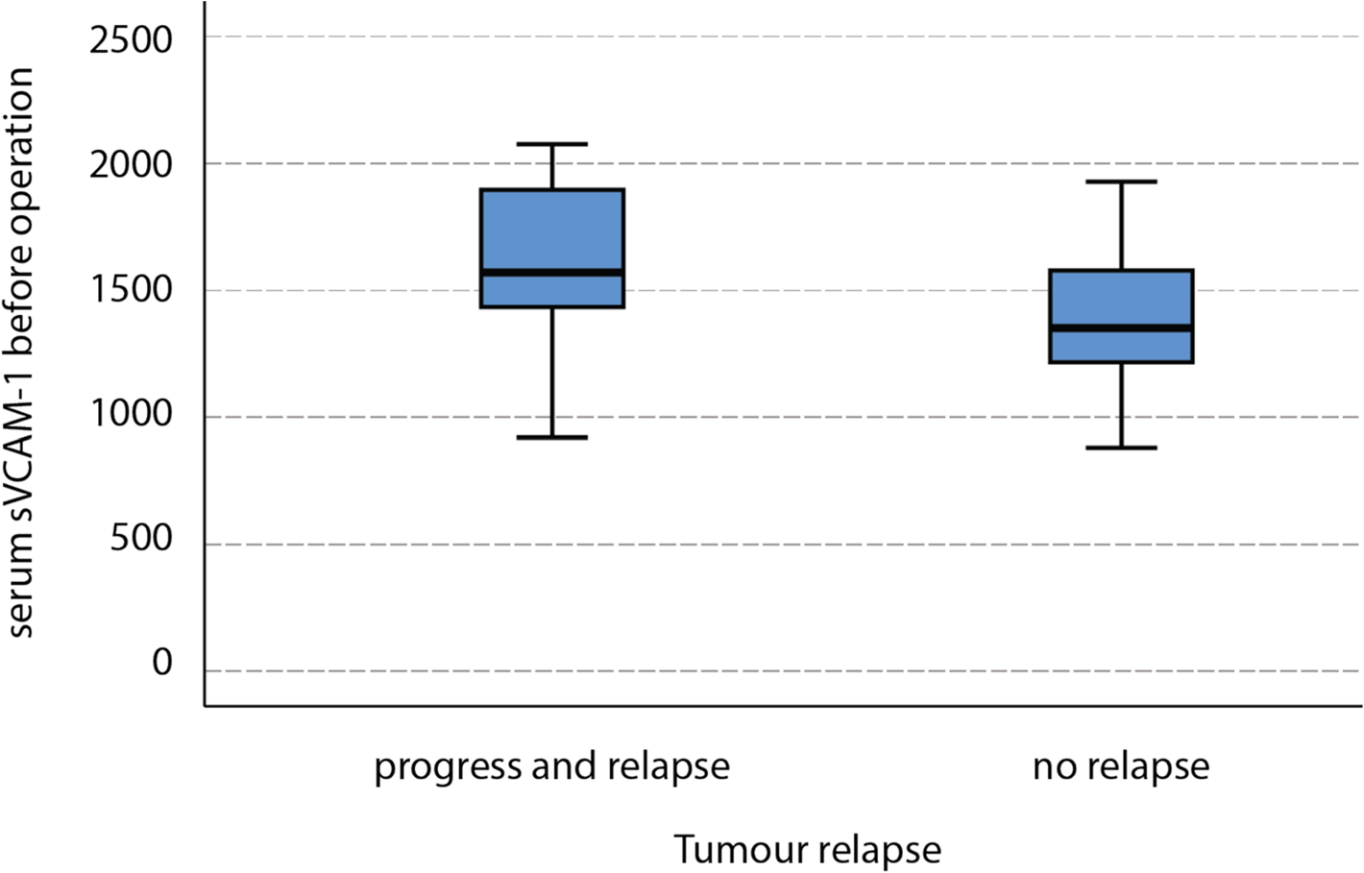
Comparison sVCAM-1 concentration in serum before operation in patients with relapse after 12 months or without relapse in the follow up period and in patients with disease progress or relapse in 12 months after the treatment

Mean serum sVCAM-1 concentration in all patients before operation was 1564.68 ± 435.65 ng/ml and mean ascites sVCAM-1 concentration was 801.84 ± 244.35. First we devided all patients in two groups. Mean sVCAM-1 concentration in the patient in group A was 1660.54 ± 417.93 ng/ml in serum and 827.92 ± 290.36 ng/ml in ascites. Mean sVCAM-1 concentration in group B was 1451.91 ± 441.15 ng/ml in serum and 771.16 ± 179.93 ng/ml in ascites. There was a significant (*p* = 0.04) higher serum sVCAM-1 concentration in group A compared with group B (See Fig. 1). There was no significant difference in ascites sVCAM-1 between the groups (*p* = 0.38). There was correlation between serum and ascites sVCAM-1 concentration in group A (*p* = 0.006) and not in group B (*p* = 0.22).

### ROC analysis and survivals

**Fig. 2:**
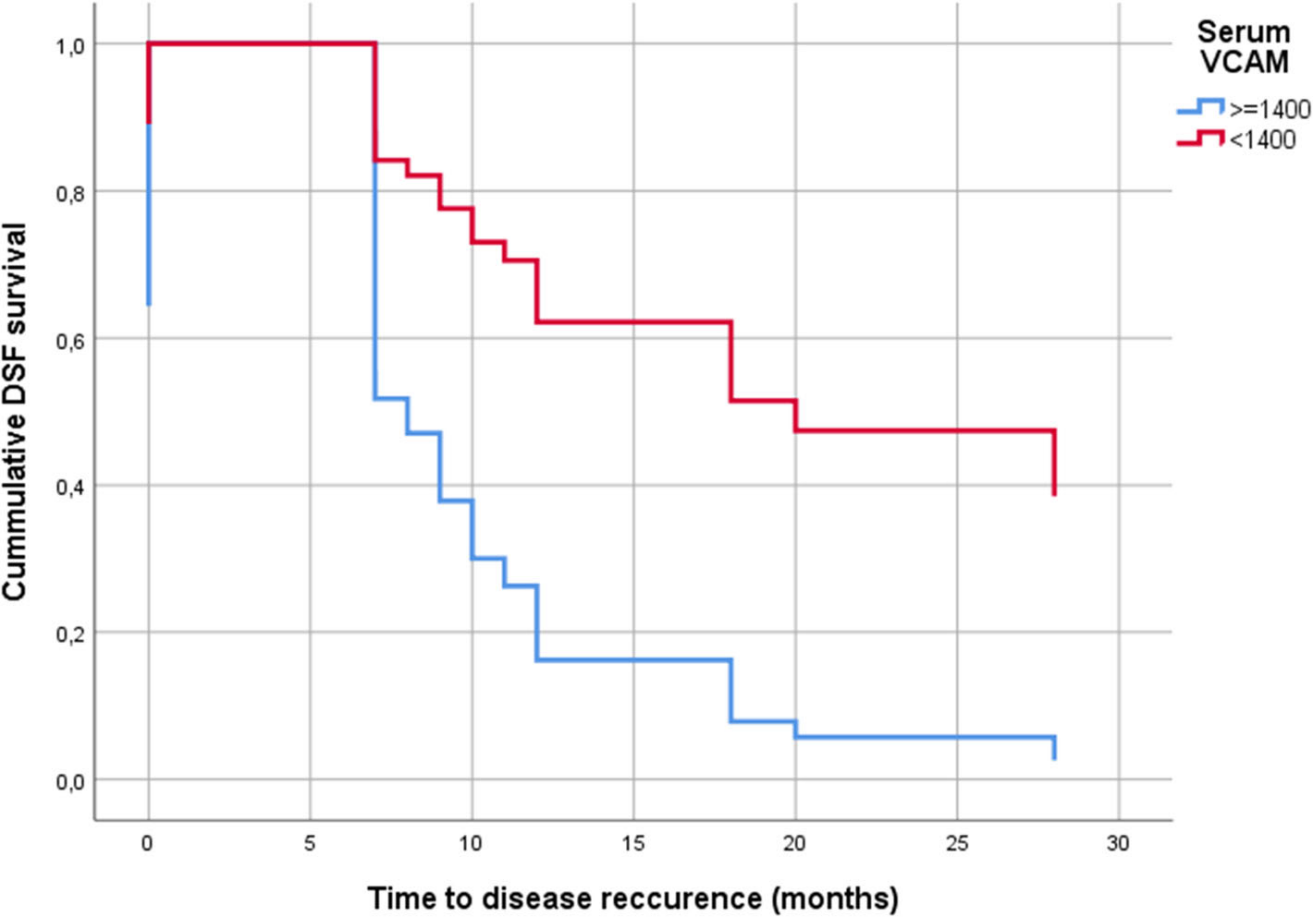
sVCAM-1 and early recurrence of the disease in patients with stage III/IV epithelial ovarian cancer (*n* = 37). Cut-off point for sVCAM-1 is 1400 ng/ml

**Fig. 3:**
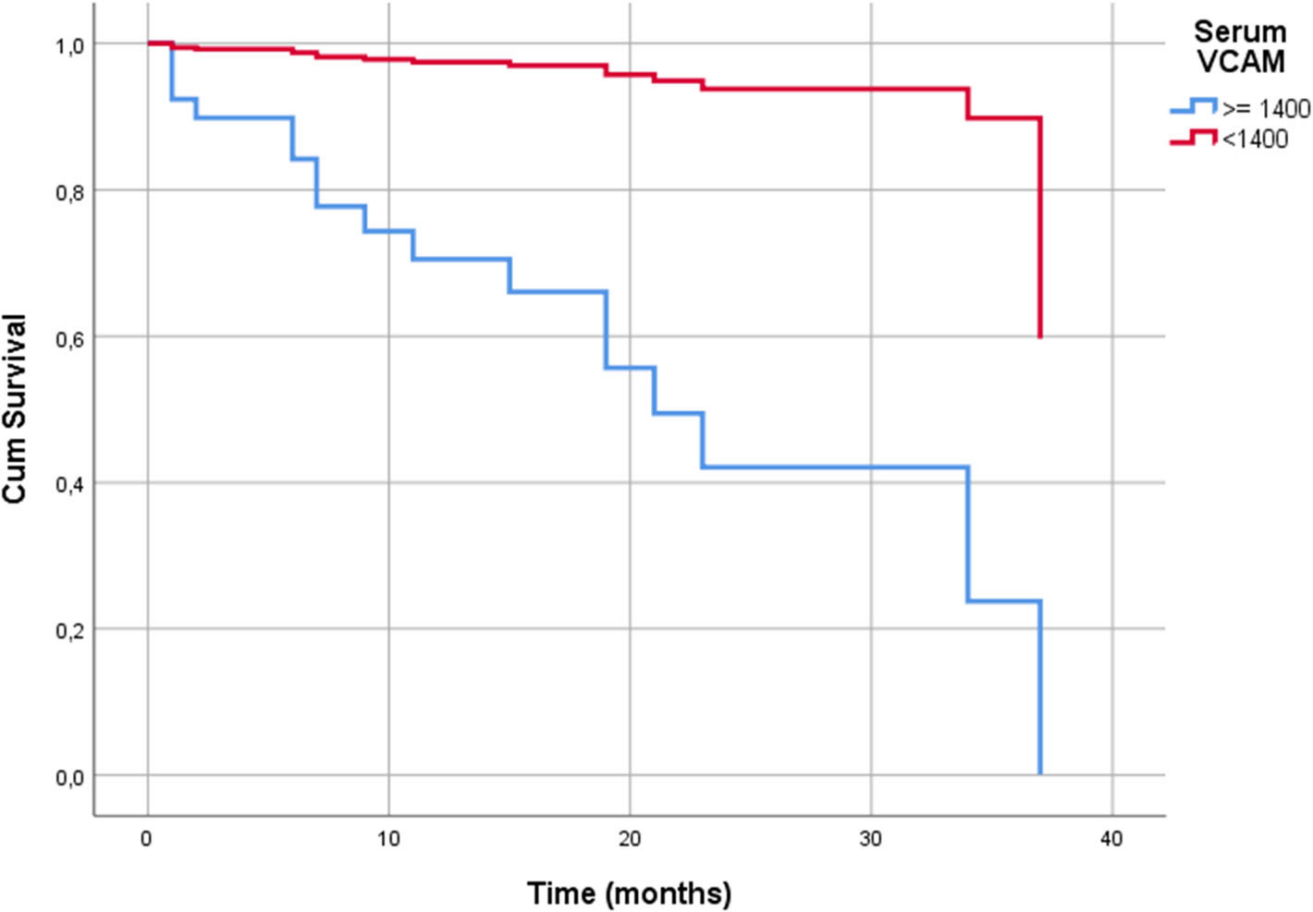
sVCAM-1 and overall survival in patients with stage III/IV epithelial ovarian cancer (*n* = 37). Cut-off point for sVCAM-1 is 1400 ng/ml

After performing ROC analysis, the maximum Youden index suggested a cut-off value of 1400 ng/ml sVCAM-1 for predicting early recurrence (< 12 months). sVCAM-1 and age resulted correlated (Spearman r = 0.32, *p* = 0.048). Adjusting for age at surgery, sVCAM-1 above 1400 ng/ml was associated with impaired DFS (hazard ratio 3.824; 95% confidence interval 1.460 to 9.374; *p* = 0.003; see Fig. 2) and impaired OS (hazard ratio 13.394; 95% confidence interval 1.693 to 105.3985; *p* = 0.014; see Fig. 3).

## Discussion

In our previous study we identified the presence of sVCAM-1 in ovarian cancer patient serum and its correlation with ascites sVCAM-1 concentration [3]. These findings further supported the idea that sVCAM-1 is measurable in serum of advanced ovarian cancer serum and reflects the ongoing ovarian cancer. In the current study, we find that there was higher pre-treatment serum sVCAM-1 in patients with early tumour progress or relapse, as compared to patients relapsing 12 months after treatment. Though we excluded patients having conditions, which could possibly interfere with sVCAM-1 concentration in plasma, patients from group A were older, so that higher concentrations of sVCAM-1 in this group should be the result of age. On calculating the threshold value of sVCAM-1 for our group of patients, we found that both age and sVCAM-1 were independent variables for early recurrence. Furthermore, the same result was also seen in overall survival of our group of patients with ovarian cancer.

VCAM-1 is a member of Ig superfamily expressed on activated endothelial and mesothelial cells. VCAM-1 expression and tumour presence association has been evidenced [6]. It has been proposed as a prognostic tumour factor in different cancers, its value being its expression of different outcome. Increased serum sVCAM-1 levels have been linked to poor survival in relation to melanoma, Hodgkin and non- Hodgkin lymphoma and breast and gastric cancer. Similar results were seen in terms of colorectal cancer [7]. In terms of renal cell carcinoma, VCAM-1 tumour cell expression is associated with better survival rate [8].

In the first study measuring sVCAM-1 serum concentrations in 15 patients with ovarian cancer it has been found that the concentration is significantly higher if patients have ovarian cancer [9]. The opposite is presented in another study, which reported lower sVCAM-1 concentration in patients with ovarian cancer compared to those with benign ovarian conditions. The likely cause of the difference may lie in patient selection, that is the one of the studies only patients with early ovarian cancer were included.^16^ Slack Davis and al. measured serum sVCAM-1 levels in ovarian cancer patient serum and state that this protein is linked to worse outcomes, whilst another recent study didn’t report any correlation between sVCAM-1 and ovarian cancer outcome, but only with metastasis presence [5, 10].

In 2013**,** Scalici et al. suggested VCAM-1 as indicator for ovarian cancer response to platinum based chemotherapy [11], and in a recent study, correlated serum sVCAM- 1 with mesothelial VCAM-1 expression in 18 patients with ovarian cancer and were unable to identify serum sVCAM-1 as a surrogate for mesothelium expression [12]. To the best of our knowledge we didn’t find any studies concerning sVCAM-1 and ovarian cancer recurrence. The role VCAM-1 in ovarian cancer is largely unclear, but it has been shown that mesothelial expression mediates tumour cell invasion [13]. In this study we measured increased levels of sVCAM-1 before operation in patients with ovarian cancer whose disease had progressed or tumour had relapsed at a very early stage, 12 months, after treatment completion. The observed increase in serum sVCAM-1 concentration could be attributed to disease biology and behaviour, as we were not able to find any correlation with tumour stage, disease grade or histological type between groups; we therefore measured sVCAM-1 from ascites as a fluid representing the local tumour microenvironment. It is an ideal media for evaluation of collected tumour cells and soluble proteins. The results from our measurements of patient’s sVCAM-1 concentrations in ascites only evidences a correlation with serum sVCAM-1 levels in the group of patients with early relapse or disease progress, but not in the group with later disease relapse. Research carried out by Slack Davis et al. attests the importance of VCAM-1in the regulation of ovarian cancer cell mesothelial invasion and metastatic progression. A possible explanation for this might be that if the tumours are more aggressive, mesothelial invasion is stronger and peritoneum mesothelial cells around the attached tumour cells are more severely affected. Peritoneum breakdown leads to easier ascites outflow with sVCAM-1 into the blood, so correlation is visible; whilst in the other group, mesothelial cell affection is slowed down and ascites transfer into the blood is limited, so sVCAM-1 correlation is not visible. Patients from this group needs longer periods of time to relapse post treatment. We also found that most patients with early relapse and tumour progress were treated with neo-adjuvant chemotherapy, whilst most patients from the group that relapsed more than 12 months post-treatment, were treated with primary cytoreductive surgery. The decision for treatment was based on radiological and laparoscopic evaluation of resectability, which is the standard pre-treatment procedure at our institution. From our findings, we can assume a preoperative concentration of serum sVCAM-1, which can be another indicator for tumour aggressiveness for predicting primary optimal cytoreduction and cancer treatment response. Ovarian cancer and cancer mediator biology are not very clear. Kong in a recent review covers the role and relevance of sVCAM-1 in inflammation and cancer, and highlights the emerging potential of sVCAM-1 as a new therapeutic target in terms of immunological disorder in cancer [9]. It has been shown that sVCAM-1 is closely associated with the progression of various immunological disorders, inflammation-associated vascular adhesion and the transendothelial migration. It seems possible that inflammation around tumours can be a crucial process for the different tumour and host behaviour, tumour cell shedding and consequently different sVCAM-1 concentrations.

The very small sample size is a limitation of this study that needs to be acknowledged. We are also well aware that other factors can influence sVCAM-1 concentration but, in our case, the exclusion criterion was the presence of a systemic disease known to affect sVCAM-1expression, both groups possibly experiencing typical inflammatory responses characteristic of cancer. To conduct multivariate analysis, we had to calculate the threshold for sVCAM-1 using the ROC analysis and maximum Youden index, being well aware, that the results stood only for our selected patients with ovarian cancer.

In correlating and evaluating serum and ascites sVCAM-1 concentration and correlation, we showed that cancer aggressiveness is reflected in serum in terms of cancer cell secretome, which is probably determinant even prior to commencement of treatment. However**,** further work needs to be done on a bigger sample size to establish whether sVCAM-1 in serum can be used in clinical practice as an early treatment outcome indicator.

Serum sVCAM-1 concentration at the time of diagnosis might be predictive of different biological behaviour and treatment resistance**,** and associated with cancer progression or recurrence. This is the first study demonstrating that higher serum sVCAM-1 concentrations in ovarian cancer patients are linked to early tumour recurrence or disease progression. Serum sVCAM-1 can be a potential tumour marker for ovarian cancer follow-up.

## Data Availability

All the data used in the analysis are obtainable on request from the principal author, Ms. Marina Jakimovska MD, PhD.

## Abbreviations

sVCAM-1: Soluble Vascular Cell Adhesion Molecule-1
FIGO: International Fderation of Gynecology and Obstetrics
